# Post-Radical-Prostatectomy Urinary Incontinence: The Management of Concomitant Bladder Neck Contracture

**DOI:** 10.1155/2012/295798

**Published:** 2012-04-26

**Authors:** Thomas King, Y. Zaki Almallah

**Affiliations:** Department of Urology, The Queen Elizabeth Hospital, University Hospitals Birmingham, NHS Foundation Trust, Edgbaston, Birmingham B15 2TH, UK

## Abstract

Urinary incontinence postradical prostatectomy is a common problem which adversely affects quality of life. Concomitant bladder neck contracture in the setting of postprostatectomy incontinence represents a challenging clinical problem. Postprostatectomy bladder neck contracture is frequently recurrent and makes surgical management of incontinence difficult. The aetiology of bladder neck contracture and what constitutes the optimum management strategy are controversial. Here we review the literature and also present our approach.

## 1. Introduction

Despite advances in surgical technique in recent years, urinary incontinence remains a relatively common complication following radical prostatectomy [1]. The true incidence of postprostatectomy incontinence (PPI) is difficult to ascertain owing to the lack of a single definition of what actually constitutes continence after radical prostatectomy. EAU guidelines define continence following radical prostatectomy as either total control with no leakage or pad usage, no pad use but loss of a few drops of urine, or use of up to one “safety” pad per day [2]. Nevertheless radical prostatectomy does represent the commonest cause of stress urinary incontinence in men [3], and it has been estimated that 14–20% of men who undergo radical prostatectomy will use absorbent pads on the long term to manage incontinence [4]. With the increasing number of radical prostatectomies currently performed, the incidence of PPI is also likely to rise [1].

PPI can have devastating effects on quality-of-life patients treated for prostate cancer and may result in considerable psychological morbidity [5]. PPI itself can be difficult to treat, and concomitant bladder neck contracture (BNC) presents an even more challenging clinical problem. The presence of a BNC itself may impact upon continence, and persistent contractures complicate the surgical management for PPI. In addition, treatment for recurrent and intractable BNC may result in de novo incontinence which then needs to be addressed.

Controversy exists as to both the underlying aetiology of bladder neck contracture and also how best to manage it. In this article we paper the literature and present our approach.

## 2. Aetiology of Bladder Neck Contracture

Bladder neck contracture, bladder neck stenosis, and anastomotic stenosis are synonymous terms and constitute a well-recognised complication following radical prostatectomy with a reported incidence of 0–32% [6–10]. Various technical and patient-based factors have been found to be associated with the formation of bladder neck contracture. However, few factors are reported with consistency between different studies and the precise aetiology remains to be firmly established.

Of the technical factors thought to play a role, the surgical approach seems to be of particular importance. Minimally invasive laparoscopic and robot-assisted laparoscopic techniques have a lower reported incidence of BNC when compared to open surgery [10–12]. Indeed in a recently published series of 4592 patients, Sandhu et al. reported surgical approach to be the strongest predictor for subsequent development of BNC with a hazard ratio of 0.11 for laparoscopic versus open surgery (*P* < 0.001) [13]. Better visualisation whilst carrying out the anastomosis allowing more accurate mucosal apposition, a continuous suturing technique, and overall reduced intraoperative blood loss have all been cited has possible reasons for the difference seen between open and laparoscopic techniques [11, 14].

Other technical factors reported to be associated with development of BNC include degree of blood loss and haematoma formation [7, 8, 13, 15], calibre of the reconstructed bladder neck [15], and early urinary retention following catheter removal [16]. Urinary extravasation has been reported as important in several studies [7, 8, 13]; however, others have found that the degree of urinary extravasation is unrelated to BNC development provided a urinary catheter is left in place until extravasation is seen to resolve on cystography [15].

Borboroglu et al. have reported significantly higher rates of post-radical-prostatectomy BNC in smokers, those with ischaemic heart disease, hypertension, and diabetes [17] prompting the hypothesis that its development may be a manifestation of microvascular disease. Similarly, a multivariate analysis by Sandhu et al. found that age, body mass index, and comorbidity in particular preexisting renal disease were predictive for formation of BNC [13]. In contrast, in a series of 650 robot-assisted laparoscopic radical prostatectomies, Msezane et al. found no difference in age and body mass index between those who developed BNC and those who did not [14]. Other patient-based factors cited as contributory include previous TURP [7, 8] and a propensity to undergo hypertrophic scarring [18]. Tumour stage [8] and Gleason score [15] do not appear to be significantly associated with BNC development.

Clearly development of BNC following radical prostatectomy is not due to one single factor but is a result of an undoubtedly complex interplay between baseline patient characteristics and technical factors. However, the surgical aim of creating a tension-free, watertight anastomosis with good mucosal apposition and minimal devascularisation of the bladder neck must be seen as the best starting point for minimizing its occurrence.

## 3. Presentation and Effect of Bladder Neck Contracture on Urinary Continence

BNC typically presents with lower urinary tract symptoms in particular reduced stream shortly following radical prostatectomy or ultimately retention of urine. Retrospective series have reported that the majority of BNCs present within 6 months following prostatectomy [18, 19]. In a series with prospective followup, Giannarini et al. reported development of BNC at a median time of 3.8 months after radical prostatectomy [20]. Investigations usually reveal a reduced Qmax and an obstructive pattern on uroflowmetry following which the diagnosis is typically made at urethroscopy with the finding of a narrowed bladder neck which will not admit a flexible cystoscope.

 Alternatively bladder neck contracture may present with or come to light during the workup for PPI. Indeed in multivariate analysis, development of bladder neck contracture has been shown to be an independent risk factor for urinary incontinence following radical prostatectomy [21]. The effect of bladder neck contracture on urinary incontinence may be several fold. Firstly, bladder outflow obstruction due to a contracture may aggravate overactive bladder symptoms and thus worsen any component of urge incontinence contributing to the patients overall symptoms. Secondly, it has been suggested that, in determining the rigidity of the anastomotic region, presence of a bladder neck contracture may impair the ability of even a preserved external sphincter contraction to close the bladder outlet efficiently [20]. Conversely by virtue of causing bladder outlet obstruction, a bladder neck contracture may mask the severity of incontinence due to sphincteric deficiency. Thus treatment for BNC can have a positive or detrimental effect on urinary continence depending on which of the above aspects is predominant in the individual patient and what procedure is used. However, the outcome in terms of continence should largely be predictable.

## 4. The Management of Concomitant BNC and PPI

The optimum treatment for bladder neck contracture is controversial, and various authors have advocated differing strategies. Often success is reported differently between studies, and direct comparison of outcomes is difficult. Overall, treatment should be considered in light of the planned strategy for dealing with PPI. Where there is no plan to intervene for PPI, the ideal treatment for BNC would be minimally invasive and have no adverse effects on continence. In this situation simple transurethral procedures are most appropriate although, owing to the recurrent nature of the problem, repeat intervention may be required. Ramchandani et al. have advocated transurethral balloon dilatation and reported a success rate of 59% after initial treatment [22]. Park et al. reported a 92% success rate at 12 months with dilatation using the Nottingham dilators followed by a 3 month self catheterisation protocol; however, 27% required more than 2 procedures [18]. The findings of Surya et al. [7] illustrate the recurrent nature of bladder neck contracture; in their series, patients were managed initially with dilatation using either the Van Buren sounds or filiform bougies and followers with those requiring dilatation more than once every 6 weeks going onto having either cold knife incision or incision with electrocautery. Only 28% were managed with dilatation alone whilst only 62% responded to a single cold knife incision with the remainder of patients requiring additional periodic dilatation. In addition this study reported de novo incontinence in all patients whose contracture was treated with electrocautery. In contrast Popken et al. reported no adverse effects on continence with their strategy of endoscopic resection using electrocautery [6]. Yurkanin et al. have used cold knife incision at 4 o'clock and 8 o'clock and report a low retreatment rate of 17% [23]. Giannarini et al. also used cold knife incision and were able to demonstrate a positive effect on continence following treatment of BNC in 90% of patients as assessed by one-hour pad testing. Of the 21 originally incontinent men in their series, 11 had become continent (less than one gram increase in pad weight) and 8 had improved on pad testing at one month following incision of their contractures [20].

 As well as self-catheterization protocols and repeat dilatations, novel strategies to mitigate contracture recurrence have been described. In a series of predominantly recurrent contractures, Eltahawy et al. report a success rate of 83% at 24 months followup using holmium laser incision followed by steroid injection [24]. Vanni et al. who describe radial incision with cold knife followed by injection with mitomycin C have found 72% success for bladder neck patency at 12 months following a single procedure [25].  Table 1 summarises the different strategies described for the endoscopic management of postradical prostatectomy BNC.

In the situation where definitive surgery for PPI in the form of artificial urinary sphincter (AUS) is planned, any BNC should be treated aggressively and a stable patent bladder neck ensured prior to AUS placement. This is to minimise the chance recurrence and the need for further endoscopic procedures which may damage the AUS once in situ or increase the chances of cuff erosion [26]. In addition, definitive treatment for a recurrent contracture may necessitate the sacrifice of continence to establish patency of the bladder outlet. As such, in these situations procedures to deal with the contracture are often combined with implantation of an AUS to restore continence.

**Figure 1:**
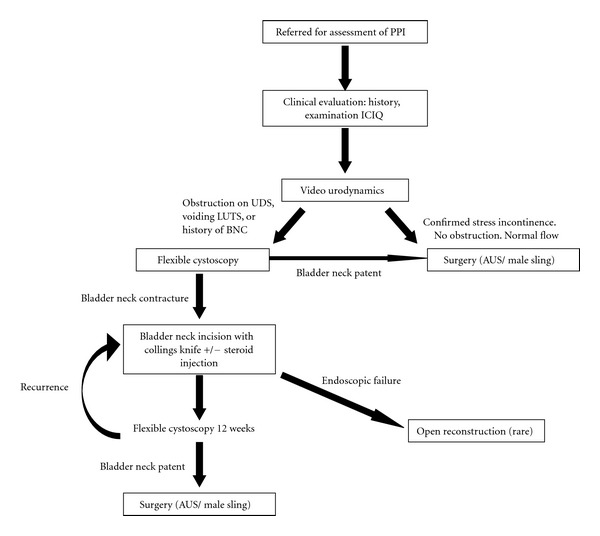
Our approach to management of PPI with concomitant BNC.

 Once again, multiple strategies have been described by different authors. Gousse et al. advocate a staged approach where aggressive transurethral incision of the bladder neck contracture at three, nine, and twelve o'clock by Collings knife is followed by AUS placement 6–8 weeks later provided a check cystocopy at 5 weeks confirms a patent bladder neck [27]. In contrast Mark et al. reported good results with synchronous Collings knife incision and AUS implantation in 26 patients [28]. Only one patient developed symptomatic BNC recurrence with the AUS in situ, and this was managed successfully with repeat incision at the time of sphincter revision. In a later paper, authors from the same institution reaffirm that a synchronous approach is successful in most situations but suggest that a staged approach may be required in patients with a history of multiple prior dilations or incisions or in the setting of long dense contractures [29]. Since BNC typically presents well before most urologists would consider surgical intervention for PPI, the majority patients with symptomatic BNC fall into the former group (having had prior procedures) by the time they are referred for surgical management of their incontinence, and, as such, practice in our tertiary centre is to carry out staged procedures (Figure 1).

Rarely, postprostatectomy BNC can be severe and entirely refractory to endoscopic measures, and these recalcitrant contractures combined with postprostatectomy incontinence represent a formidable challenge in terms of management. Essentially two strategies have been proposed to avoid long-term catheterization or urinary diversion: placement of a UroLume stent combined with AUS or open surgery to reconstruct the bladder neck again combined with AUS to restore continence.

Elliott et al. reported good results using UroLume stents followed, after a 3 month interval, by AUS placement in a series of 9 patients with recurrent contractures and severe stress incontinence. 88% were satisfied with the procedure, mean pad use declined from 6.5 to 0.7 per day, and within the 17.5 month followup no reoperations were required on the AUS [30]. One patient did, however, develop stent ingrowth which required placement of second overlapping stent. Anger et al. have also used this strategy following the failure of endoscopic management but proposed a shorter interval between stent placement and AUS implantation of only 4–6 weeks in order to minimise the period spent with total incontinence. In their series of 8 patients, stent obstruction due to ingrowth occurred in two patients at 4 and 6 months, respectively, and was successfully managed using a flexible ureteroscope and holmium laser resection of the hyperplastic tissue [29]. However, in a later study from the same institution with longer followup, Borawski and Webster report that 50% of patients managed with UroLume stent combined with AUS required an average of 2.25 procedures for stent ingrowth over 37 months. In addition 11 patients (27.5%) required a total of 18 AUS uncouplings to facilitate endoscopic treatment, and the risk of cuff erosion was higher (35% versus 10%) in those requiring treatment for stent ingrowth [26].

It has been reported that the rate of symptomatic tissue ingrowth with urethral stents in general approaches 25% [31]. In the setting of recurrent postradical prostatectomy BNC, the 5-year cumulative incidence of treatment failure following insertion of a single stent has been estimated at 50% [32]. This, together with other problems such as haematuria, stent migration, encrustation, and perineal pain, means that the role of stents must be seen as extremely limited in a setting where additional procedures to deal with stent complications are themselves compounded by the presence of an artificial urinary sphincter.

The alternative to UroLume stents in fit patients with good life expectancy and cancer control is open surgery to excise the stenosed bladder neck together with adjacent scar tissue and fashion a new anastomosis; again this is normally combined with AUS implantation to restore continence. Various approaches have been described including perineal [33] and combined abdominoperineal [34] with synchronous [34] or delayed [33] placement of AUS. Some authors [35, 36] advocate pubectomy to facilitate exposure and allow for a tension-free anastomosis whilst others have found this unnecessary [33]. A variety of reconstructive techniques have been used with similar success; however, owing to the rarity of the need for such interventions, the numbers in all published series are necessarily small [33–37]. Indeed because these patients are complex, no single technique is likely to be applicable in all cases, and instead an individualised approach is required. This is illustrated by Wessells et al. who describe a series of four patients with obliterative contractures each requiring a different technique of reconstruction ranging from simple end-to-end anastomosis to onlay urethroplasty using full-thickness penile skin graft with rectus muscle flap for graft coverage [35]. In experienced centers, the long-term success from such procedures is around 70% [37].

## 5. Concomitant BNC and the Male Sling

The insertion of a synthetic suburethral male sling for the treatment of PPI is a relatively new procedure but is gaining worldwide popularity as a less invasive alternative to implantation of an AUS which also maintains spontaneous urethral voiding [38]. Sling devices seem to offer the best results in men with mild-to-moderate stress incontinence [39]. However, the global experience is still limited and more long-term results are awaited. Consequently experience of patients with concomitant BNC undergoing male sling is even more limited. Our standard practice is to attempt to identify patients with BNC prior to the insertion of a male sling. Patients with significant contracture will need bladder neck incision with Collings' knife with the possibility of upgrading their PPI. This itself may lead to a change in the overall management plan for their urinary incontinence. In our experience, patients with concomitant BNC who initially appear to be suitable for the insertion of male sling often need the AUS once their BNC has been treated. However, in patients with mild BNC, it can be argued that synchronous treatment of BNC with insertion of a male sling may be appropriate [40]. Treatment of mild bladder neck stenosis is unlikely to change the grade of PPI, and the possibility of those patients having significant recurrence of BNC, which will require treatment in the future, is likely to be small.

## 6. Our Approach

Our unit has ran a specialist service dedicated to the management of PPI for a number of years, and the framework of our basic approach is illustrated in Figure 1. All patients receive a thorough initial clinical evaluation in terms of history, clinical examination, and ICIQ scoring. Subsequent investigation centres on high-quality video urodynamic studies to demonstrate stress incontinence and assess bladder capacity, any coexistent detrusor overactivity, or evidence of bladder outflow obstruction. Following this, patients found to have evidence of obstruction on urodynamics; those with voiding LUTS or a history of prior bladder neck contracture precede to flexible cystoscopy for further assessment and management as illustrated. Practically speaking almost all our patients undergo flexible cystoscopy as it also provides an opportunity to evaluate the sphincter condition; however, a small number with severe stress incontinence and no evidence of obstruction precede directly to surgery following urodynamic assessment.

## 7. Conclusion

The management of bladder neck contracture in the presence of PPI is challenging. Most patients can be successfully managed endoscopically, and cases requiring open excision and reconstruction are fortunately rare. Treatment of bladder neck contracture in the setting of mild PPI can be managed with conservative steps in the form of dilatation followed if necessary by intermittent self-catheterization to maintain patency. However, if the PPI warrants surgery, any concomitant BNC must be treated aggressively, and a stable, patent bladder neck should be ensured prior to placement of any prosthesis in order to avoid complicated recurrence.

## Figures and Tables

**Table 1:** Endoscopic management of postprostatectomy bladder neck contracture.

Authors	Year	*n*	Technique	Results
Surya et al. [7]	1990	18	Dilation with sounds followed by cold knife incision unsuccessful after 6 months	28% managed with dilation alone Single CKI effective in 62%
Ramchandani et al. [22]	1994	27	Transurethral balloon dilation	Successful in 59%
Popken et al. [6]	1998	15	Electrocautery resection	53% required >1 procedure
Yurkanin et al. [23]	2001	36	Cold incision at 4 and 8 o'clock	Repeat procedure required treatment in 17%.
Park et al. [18]	2001	26	Dilation over wire followed by CISC for 3 months	92% managed with successfully 27% require ≥2 dilations in 12 months
Giannarini et al. [20]	2007	43	Dilation followed by cold incision at 4, 8 and 12 o'clock	7% managed with dilation only 26% recurrence after incision
Eltahawy et al. [24]	2008	24	Holmium laser incision at 3 and 9 o'clock + steroid injection	83% patent bladder neck at 24 months 29% require 2nd procedure
Vanni et al. [25]	2011	18	Cold incision + mitomycin injection	72% patent at 12 months after single procedure

## Abbreviations

PPI: Postprostatectomy incontinence
BNC: Bladder neck contracture
AUS: Artificial urinary sphincter
BNI: Bladder neck incision.
